# “*Don’t* Mind the Gap!” Reflections on Improvement Science as a Paradigm

**DOI:** 10.1007/s10728-017-0353-7

**Published:** 2017-11-17

**Authors:** Trenholme Junghans

**Affiliations:** CRASSH, University of Cambridge, 7 West Road, Cambridge, CB3 9DP UK

**Keywords:** Improvement science, Conceptual metaphor, Complexity, Pragmatism, Paradigm, Incommensurability

## Abstract

Responding to this issue’s invitation to bring new disciplinary insights to the field of improvement science, this article takes as its starting point one of the field’s guiding metaphors: the imperative to “mind the gap”. Drawing on insights from anthropology, history, and philosophy, the article reflects on the origins and implications of this metaphoric imperative, and suggests some ways in which it might be in tension with the means and ends of improvement. If the industrial origins of improvement science in the twentieth century inform a metaphor of gaps, chasms, and spaces of misalignment as invariably imperfect and potentially dangerous, and therefore requiring bridging or closure, other currents that feed the discipline of improvement science suggest the potential value and uses of spaces of openness and ambiguity. These currents include the science of complex adaptive systems, and certain precepts of philosophical pragmatism acknowledged to inform improvement science. Going a step further, I reflect on whether or not these two contrasting approaches within improvement science should be treated as incommensurable paradigms, and what each approach tells us about the very possibility of accommodating seemingly irreconcilable or incommensurable approaches within improvement science.

A newcomer to the field of improvement science might be forgiven for thinking that healthcare improvement is fundamentally a project of gap closure: from bridging the iconic “quality chasm” proclaimed by the landmark Institute of Medicine 2001 report [14], to closing the “implementation gap” between what is *known* about effective care and what is actually *done* in practice [10], the terrain of improvement science might appear to the newcomer to be textured by dangerous crevices and gullies, and even the occasional abyss. In keeping with this challenging topography, the discipline’s mandate and mission might be construed as a matter of bridging gaps, fording chasms, and correcting improper alignments. In sum, one might reasonably regard “mind the gap!” to be a fitting exhortatory motto for improvement science.

In response to this special issue’s goal of bringing new disciplinary perspectives to constructively bear on improvement science, I would like to critically reflect on this imperative to “mind the gap”: from where does this imagery come, when and how does it serve the ends of improvement science, and when and how might it be less conducive to the aim of improving healthcare? What might be lost or excluded when we regard gaps as always and invariably dangerous, and spaces of misalignment as necessarily imperfect? When might benefits be had if we *do not* “mind the gap”? In pursuing this thought experiment I will draw on insights from anthropology, history, and philosophy to suggest how the pervasive mandate to “mind the gap” might be in tension with some of the core philosophical precepts on which improvement science is based. In the process I will also suggest ways in which the same conceptual schemes and habits of thought that lend coherence and urgency to the imperative might also inform an impulse to construe improvement science in narrow paradigmatic terms that can unnecessarily exclude other disciplinary perspectives.

## Framing the Problem: The “Quality Chasm” as Conceptual Metaphor

At first glance, thinking about problems in healthcare in terms of gaps and chasms, and solutions in terms of bridges, crossings, and other processes and devices of re-alignment and closure, might seem self-evident to anyone with even a passing familiarity with improvement science. Beginning in the 1990s a series of studies and reports revealed staggering and theretofore unrecognized shortcomings in the health care systems of industrialized countries, such that it was estimated that on average one in ten hospitalizations resulted in preventable harm, including death [32]. A study in 2000 found that on average it took 17 years to get 14% of results from randomized controlled trial studies into practice [3]. This slow rate of uptake was further exacerbated by the staggering rate at which new findings from medical studies were being published: medical knowledge was proliferating at an exponential rate, and far outstripping the capacity of medical professionals to access it, let alone put it into practice [8]. The extent and depth of the problem was further underscored by the 2003 finding that in a full 45% of cases Americans did not receive the care that would be indicated by the best available evidence [23]. Hence to a significant extent, the epidemic of unsafe, poor quality healthcare that was coming into focus was being diagnosed in terms of failures to implement what was recognized to be best practice, be it clinical, managerial, or organizational practice.

Today our thinking about these urgent problems has come to be inseparably bound up with particular images and metaphors. Arguably the most iconic and influential of these was proclaimed in 2001 with the publication of *Crossing the Quality Chasm: A New Health System of the 21st Century*. On its first page the report proclaimed the magnitude of the disjuncture alluded to in its title: “between the health care that we now have and the health care that we could have lies not just a gap, but a chasm” [14]. The abysmal dimensions of the problem were intimated by the report’s cover image, a somewhat abstract rendering of what might be variously construed as rent fabric, a cleft structure, or a seismologically ruptured cross-section of earth. The implication seems to be that gaps, spaces in-between, and states of misalignment or disjuncture are sources of unabated risk and danger, and require reparation by means of suturing, realignment, or bridging.

We might think of the quality chasm and the gaps that implementation science seeks to close as variations on an overarching conceptual template, or what cognitive linguists refer to as a *conceptual metaphor*. Conceptual metaphors are common linguistic phenomena whereby one concept is understood in terms of another [18]. Far from being superficial features of language, or ornamental accessories characteristic of poetic expression, conceptual metaphors are fundamental to human language and cognition, and facilitate one’s ability to make sense of a new concept or experiential domain in terms of another that is more familiar. This mapping across conceptual domains facilitates understanding and logical inference, and typically involves understanding an abstract concept in terms of another concept that is more concrete. A paradigmatic example provided by Lakoff and Johnson is “time is money”, an association which lends coherence to a host of other formulations and inferences, such as “wasting time”, “living on borrowed time”, repaying a debt to society by “serving time”, or even something being “worth one’s while”. Conceptual metaphors are frequently implicit rather than explicit, and are often so thoroughly embedded in a culture’s language, thought, and norms of behavior that they may go completely unnoticed, and assume a cast of natural givenness or inevitability.

Because of the role played by conceptual metaphor in conditioning thought and action, it follows that reflection on the “metaphors we live by” (the title of Lakoff and Johnson’s influential 1980 book on the subject) can support innovative thinking and creative problem solving. To pursue this insight in relation to improvement science generally and with respect to the “quality chasm” in particular is in no way to question the importance of putting evidence into practice, or the value of efforts to promote the uptake of research findings. Rather, reflection on the conceptual metaphors that guide a great deal of thinking in improvement science can alert us to their possible limitations and stimulate new insights.

In what follows I will describe how the industrial origins of improvement science in the mid-twentieth century inform a conceptual metaphor of gaps, chasms, and spaces of misalignment as *invariably imperfect, defective, or potentially dangerous*. Over the last two decades these industrially-inflected associations have been augmented and in some ways challenged by precepts associated with complexity science and post-industrial forms of production and management. These developments inform an alternative conceptual scheme which evinces a much greater tolerance for uncertainty, non-alignment, and the “spaces in between”. Such an ethos or sensibility arguably has an important role to play in the pursuit of improvement, not least of all as it can powerfully inform receptiveness to alternative disciplinary approaches. Nonetheless such an orientation tends to be disparaged and marginalized as being at odds with the rigor, certainty, and epistemic probity deemed to be the condition for—and the mark of—serious scientific endeavor.

My interrogation of the mandate to “mind the gap!” is intended to serve a double purpose. First, such an exercise will suggest how gaps left open, tentative definitions, and imperfectly resolved boundaries might in some cases enhance rather than impede the aims of improvement science. Second, and perhaps more appositely for the subject of this special issue, I have a hunch that the same conceptual scheme that supports a categorical insistence on gap closure, tidy alignment, and the eradication of ambiguity also strongly informs an impulse within improvement science to cast a suspicious eye upon disciplinary approaches and knowledge practices other than those most closely associated with the natural sciences and clinical research. In other words, to the extent that the imperative to “mind the gap” maps onto concerns with paradigmatic integrity, it provides a fittingly indigenous vehicle for engaging some of the larger issues and questions posed by the topic of this special issue.

## Contours of the Quality Chasm as Conceptual Metaphor: Industrial Origins

The fact that improvement science has its origins in industrial manufacturing in the mid-twentieth century [7] provides a useful backdrop for thinking about some of the habits of thought and inference fostered and supported by the “quality chasm” as conceptual metaphor. “Quality” in industrial manufacture was initially bound up with standardization and the minimization of variation, and was closely related to notions of “fitness for purpose” or “conformance to requirements and “one best way” of doing things [2]. Because this original rendering of quality improvement arose in the context of industrial production as epitomized by the Fordist assembly line, it is easy to see why problems and inefficiencies could be readily figured in terms of gaps, spaces of disjuncture, disarticulation or misalignment. In this industrial regime problems would certainly ensue if factors of production were not properly aligned, be those factors mechanical cogs; undisciplined or refractory workers; or weak links in chains of supply or communication. What’s more, the fact that statistics was recognized early on as a powerful tool in the identification and correction of normative deviations and deficits in the production process reinforced a vision of mathematically achievable precision in the minimization of variation and the remedying of inefficiencies.^1^ This valorization of alignment, uniformity and the minimization of deviation was continuous with both a strong belief in the power of science to enhance efficiency and rationalization, and a mid-century cultural ethos that put a high premium on discipline and conformity as civic virtues unto themselves. This vision of the social and economic bounties presumed to flow from mathematically reckoned norms and processes was boldly proclaimed in 1931 by Walter Shewhart, one of the early pioneers of improvement science in industry:

The object of industry is to set up economic ways of satisfying human wants and in so doing to reduce everything possible to routines requiring a minimum amount of human effort. Through the use of the scientific method, extended to take account of modern statistical concepts, it has been found possible to set up limits within which the results of routine efforts must lie if they are to be economical. Deviations in the results of a routine process outside such limits indicate that the routine has broken down and will no longer be economical until the cause of trouble is removed [6].

Here we can see the mandate to “mind the gap” in the form of precisely specified norms and targets; an aspiration to complete routinization; and the subordination of other concerns to the interests of economization. Each of these comprises a form of gap closure in the sense that future-oriented behavior is to be guided by rigorously pre-specified expectations, and outcomes are to be judged according to the degree of correspondence, or tightness of the fit, between them.

This aspiration to the ideals of uniformity, complete routinization and standardization in industry would come to be qualified and modulated in the 1980s and 1990s, especially in association with what came to be known as the “quality revolution” in industry in the United States and Western Europe [9]. Spurred in large part by fear of declining competitiveness in the face of surging Japanese industrial and economic might, the “quality revolution” involved the adoption of more flexible and less hierarchical forms of workplace organization and management, and entailed a vastly expanded notion of “quality”: no longer was quality narrowly associated with *consistency* (of product, process, or outcome); in this period quality came to be increasingly associated with less tangible or readily quantifiable attributes, such as workplace culture, and the attitudinal and dispositional attributes of workers and managers alike [29, 33]. While continuous with the original industrial rendering of “improvement” in name and core methodological precepts, this newer version also differed in significant ways: its softer, more self-reflexive orientation found expression in a strong penchant for organic and even spiritual imagery over the mechanical. Additionally, as “quality” came to be recursively ascribed to *almost anything* bearing on an organization’s aims or activities (including the processes by which an organization would monitor its own quality),^2^ the pursuit of improvement became more urgent, but also significantly more diffuse and open-ended. Improvement methodologies were expanding dramatically, both in terms of the disciplines they drew upon, and in terms of the range of objects (and subjects) to which they were being applied [29, 33]. This softer version of quality, together with its expanding scope of application, was part of a larger dispositional shift, characterized by Nigel Thrift as “the emergence of a structure of feeling in Euro-American societies which frames the world as complex, irreducible, anti-closural” and that yields “a much greater sense of openness and possibility about the future” [29]. In this context improvement experts such as Deming were unabashedly styled as “gurus” [7], and a number of competing improvement methodologies came to be aggressively marketed as proprietary brands.^3^

Improvement science in healthcare came of age in the shadow of the quality revolution of the 1980s and 1990s,^4^ and today seems to bear the imprint of two rather different sensibilities—one that embraces ideals of precision and mastery, and accords with the mandate to “mind the gap”, and another that doesn’t just cope with openness and spaces of indeterminacy, ambiguity, and unboundedness, but actively embraces them as resources unto themselves and sources of potential value. While recognizing that each of these contrasting stances and sensibilities is suited to different ends, it is nonetheless worthwhile to dwell on the contrast between them, as I believe that their uneasy coexistence has something useful to tell us about the openness or otherwise of improvement science to alternative disciplinary approaches.

Consider the following definition of “quality of care” as the object of improvement provided by renowned British patient safety expert Charles Vincent:

[Quality of care] is defined as the proportion of potential health gain actually delivered by a healthcare organization for its set of patients. The essential idea is that quality reflects the gap between what can be achieved and what actually happens. When the gap is small, quality is good; when the gap is large, quality is poor [32].

Contrast this with a definition of quality improvement by American academic physicians Paul Batalden and Frank Davidoff:

Many in healthcare today are interested in defining “quality improvement”. We propose defining it as the combined and unceasing efforts of everyone—healthcare professionals, patients and their families, researchers, payers, planners and educators—to make the changes that will lead to better patient outcomes (health), better system performance (care) and better professional development (learning). This definition arises from our conviction that healthcare will not realize its full potential unless change making becomes an intrinsic part of everyone’s job, every day, in all parts of the system. Defined in this way, improvement involves a substantial shift in our idea of the work of healthcare, a challenging task that can benefit from the use of a wide variety of tools and methods… [4].

Vincent’s formulation recapitulates the mandate to “mind the gap” in two senses: first, with the explicit definition of quality as the perfect alignment between the potential and the actual; and second, with the crisp, formal affirmation of the *very possibility* of a precise and singular definition. In contrast, Batalden and Davidoff provide a definition which, by virtue of its all-inclusive open-endedness, is almost an anti-definition. Affective commitment and ceaseless striving become primary ends unto themselves, and improvement is cast as emergent, with no predefined limits or finite specifications.

As it happens, the latter position taps into an altogether different conceptual schema than that which is overtly proclaimed by *Crossing the Quality Chasm*. This alternative schema derives from complexity theory,^5^ and suggests that in some cases the “spaces in between” need not be dangerous spaces of deficits and defects; they might instead contain untapped resources and unrealized potential. To extend the “gap” metaphor, such an approach doesn’t just seek to *close* spaces of misalignment and uncertainty; it also mines them as potential sources of new knowledge and possibility. A brief discussion of this alternative approach and the debate it provoked will help to crystalize some of the less obvious but nonetheless important issues at stake in debates about the inclusiveness and internal coherence—or otherwise—of improvement science as a paradigm.

## The Chasm Reconsidered: Complexity as a New Conceptual Metaphor for Improvement Science

The enlistment of complexity theory into the conceptual repertoire of improvement science can be seen as part of what has been described as a “complexity turn” in the social and cultural sciences since the 1980s [31]. The application of complexity-inspired thinking to healthcare improvement was announced *sotto voce* as it were, in a manifesto-type statement that appears as an appendix to *Crossing the Quality Chasm*. The appendix, entitled “Redesigning Health Care with Insights from the Science of Complex Adaptive Systems”, was by Paul Plsek, an American management consultant and quality expert trained as a systems engineer. Around the time of the report’s 2001 release, Plsek and co-author Trisha Greenhalgh also published a series of four articles in the *British Medical Journal* (BMJ) making the case for a complexity-informed approach to quality improvement in healthcare. Together the appendix of *Crossing the Quality Chasm* and the BMJ articles deployed complexity theory to advance an approach to improvement that differs in significant ways from the original industrial version, and effectively subverts the imperative to “mind the gap”.

The appendix of *Quality Chasm* begins with a distinction between systems that are mechanical (such as fans and thermostats) and systems that are complex and adaptive (such as healthcare).^6^ Unlike the mechanical systems characteristic of Newtonian science, complex systems are held to be characterized by nonlinearity, the production of novelty, and a tendency toward inherent order, self-organization, and the co-evolution of elements [14]. Plsek and Greenhalgh claim that healthcare systems are currently designed and managed on faulty principles, namely those that only properly apply only to mechanical systems (such as the industrial assembly line). The conclusion is that the Newtonian “clockwork” universe, and with it healthcare systems, need to be re-envisioned and redesigned in far less mechanistic terms.

Newton’s “clockwork universe”, in which big problems can be broken down into smaller ones, analysed, and solved by rational deduction, has strongly influenced both the practice of medicine and the leadership of organisations. For example, images such as the heart as a pump frame medical thinking, and conventional management thinking assumes that work and organisations can be thoroughly planned, broken down into units, and optimised. But the machine metaphor lets us down badly when no part of the equation is constant, independent, or predictable. The new science of complex adaptive systems may provide new metaphors that can help us to deal with these issues better [26].

Healthcare practitioners and policy makers were urged to move away from received paradigms of mastery and certainty, and to relinquish aspirations of rigid planning, command, and control. In addition to being prompted to attend to their language and choice of metaphor, they were also entreated to embrace paradox, dwell with uncertainty, and give free rein to a new and emergent sense of creativity.

In complex systems, unpredictability and paradox are ever present, and some things will remain unknowable. New conceptual frameworks that incorporate a dynamic, emergent, creative, and intuitive view of the world must replace traditional “reduce and resolve” approaches to clinical care and service organization [26].

The emphasis placed on language use and the affordances of metaphor and paradox in particular is striking, and might be thought of as a revisioning of gaps and chasms at the level of *linguistic form*: As has been suggested, metaphor operates by juxtaposing one kind of entity into a conceptual domain with which it is not typically associated (“an avalanche of numbers”; “a stampede of shoppers”), thereby giving rise to new associations and possible meanings. On this view spaces of misalignment—or gaps and disjunctures between concepts—can give rise to novel imaginings, and ought to be cultivated as assets rather than eradicated as imperfections.^7^

Taken in its entirety, *Crossing the Quality Chasm* performs a careful balancing act: the title proclaims the need to bridge a dangerous chasm, while the appendix refigures and revalues the chasm altogether. And curiously, in spite of the strong distinction asserted in the appendix between incommensurable scientific paradigms—the Newtonian with its emphasis on order and clear definition, and the paradigm of complexity science, with its embrace of the affordances of the paradoxical and the unknown—the report as a whole *makes no effort to reconcile the two*. In this way the report exemplifies (or enacts) the same comfort with paradox and uncertainty which the appendix espouses in the name of complexity theory. By juxtaposing two antithetical paradigms, and refusing to acknowledge—let along resolve—the tension between them, *Crossing the Quality Chasm* tacitly refuses its titular mandate to bridge, harmonize, align, or reconcile.

## “Let them Eat Complexity”: The Language (Politics) of Improvement Science

It is hardly surprising that these complexity-inflected precepts and prescriptions would rub many in the clinical science community the wrong way. In an acidly worded letter to the editor entitled, “Let them eat complexity: the emperor’s new toolkit”, one BMJ reader pilloried “complexity theory as metaphor” as recapitulating modish intellectual trends associated with postmodernism, which he claimed to be romantic and antirational, and hence antithetical to the values of scientific method that ought to rightfully guide both medicine and improvement science. The author proclaimed the complexity prescriptions to be “intellectual snake oil” peddled by “healthcare administration faddists” who endanger honest scientific inquiry [27]. *That* “complexity as metaphor” might be perceived as a glib and frivolous affront to the principles of clinical science is not particularly surprising; *why* these prescriptions could provoke such a strident and categorical response, on the other hand, might be less obvious, and suggestive for considering the openness or otherwise of improvement science to other disciplinary approaches.

In his antipathy to complexity as “misapplied metaphor” the author of *Let them eat complexity* is heir to a long and august lineage of anti-rhetorical crusaders who regard metaphor as epistemologically and morally suspect. An array of cross-disciplinary scholarship attests to the fact that with advances in science and mathematics in the seventeenth century came new ideas about the relationship between language and the world, and “a quickened impulse toward symbolic precision”. This entailed modeling language and thought on a “mathematicized” ideal, and an assault on rhetoric and other language forms perceived to harbor or encourage ambiguity [19]. Thus John Locke railed against figurative language that has no purpose other than to “insinuate the wrong *Ideas*, move the Passions, and thereby mislead the Judgment” as “perfect cheats” [19]. According to such thinking, ambiguous language, epitomized by rhetoric and metaphor, became a locus of both moral and epistemological danger. The ensuing “flight from ambiguity” [19] was highly moralized, and part of a broader shift that accompanied the rise of modern science: the idea—and ideal—that language could and should describe the world in a completely neutral, transcriptive manner:

The general feature of Enlightenment that contravenes inherited rhetorical tradition is the development, in various domains, of a mode of discourse conceived as *neutral, non*-*positional, and transparent*. Nowhere is this tendency more apparent than in the emergence of science, the most powerful innovation of the post-Renaissance world…. From its beginning, science relied on the convention of a putatively true and undistorted—that is, arhetorical—depiction of natural states of affairs [5].

According to this view, language is to be stripped of any ornaments that might corrupt a normatively desirable correspondence between of word and world:

The Royal Society in seventeenth-century England… sought to cleanse language of the rhetorical excesses that would interfere with a proper, transparent relationship to the objects of denotation, a goal that appears to persist in, for example, logical positivism of the early twentieth century [15].

In sum, modern constructs of objectivity and science have been crucially bound up with a language ideology that posits the possibility and epistemological propriety of language conceived as a “neutral, non-positional, and transparent” medium. Language ideologies refer to beliefs held concerning the nature of the relations among language and the world, and what are believed to be morally and epistemologically appropriate ways of using language. As such they are implicated in judgments about language users and the claims they make [16, 34]. It is widely held by sociolinguists that Euro-American common sense notions of language tend to correspond to the Locke-inspired view off language described above. According to this view language is regarded as “an autonomous system representing or corresponding to a separately accessible social world” and “naming and propositionality are the key functions of languages, which are seen as bounded systems” distinct from the social worlds they describe [11]. As familiar as this view of language might be, it is not without its critics. What’s more, and contrary to the claims of the disgruntled BMJ reader, skepticism about language as a neutral and transcriptive medium of representation need not issue from postmodernism or academic deconstructionism; nor need it call into question the objectivity of the external world. Rather, such skepticism might simply assert that our descriptions of the world are inevitably partial and selective with respect to both the perspective of the observer and the phenomenological complexity of the object being described. Put differently, the map is not the territory. And as will be seen below, recognition of *this* gap—the disjuncture between word and world—is crucial to both the theory and the practice of improvement science, especially as informed by philosophical pragmatism.

Philosopher Nelson Goodman’s 1972 essay, “Seven Strictures on Similarity” provides an apt point of entry for considering why any categorical insistence on gap closure might be considered to be epistemologically dangerous, and how this caution informs improvement science. “Similarity”, Goodman claimed with rhetorical flourish, “is insidious”:

Similarity, ever ready to solve philosophical problems and overcome obstacles, is a pretender, an impostor, a quack. It has, indeed, its place and its uses, but is more often found where it does not belong, professing powers it does not possess [12].

Translated into a more prosaic idiom, Goodman’s point was that ascriptions of similarity can be epistemologically and philosophically fraught because any reckoning of similarity will always be partial, and consequent upon selective attention to certain aspects of objects under consideration, and disattention to other aspects:

As Nelson Goodman (1972) famously pointed out, similarity alone might be taken to be an empty explanatory construct: any two things can be similar or dissimilar as you like, depending on the respects in which their similarities or dissimilarities are described. The pigeon outside my window, the chair I’m sitting on, and the computer on my desk share numerous similarities with respect to their closeness to me, and their distance from the sun, and so on. They all share numerous dissimilarities in respect of their animacy, or lack of same, or with respect to whether they are actually in my office or not, etc. Unless we specify the *respects* in which things are said to be similar, the act of saying that they *are* similar is an empty statement [13].

Going a step further, far from necessarily being an impediment to effective intervention in the world, recognition of the inescapably partial nature of our representations of the world is crucial to improvement methodologies. In the words of organizational theorist Haridimos Tsoukas:

Our descriptions of the world are inherently incomplete. There always are more ways of thinking about the world than those in use at any point in time…. Any phenomenon can be represented through other forms that may not yet have been stated or invented—indeed that is what is assumed by, say, efforts to continuously improve quality… What we have available is a finite representation of something, never a complete one… The thing can always be presented in more ways than we already know; the thing will always hold more appearances in reserve [30].

In this observation Tsoukas is nodding in the direction of the philosophical tradition out of which improvement science grows: American philosophical pragmatism as it developed around the turn of the twentieth century through the work of Harvard-based philosophers C.S. Peirce, William James, John Dewey, and C.I. Lewis.^8^ Two influential pioneers of improvement science, Walter Shewhart and W. Edwards Deming, were each powerfully influenced by pragmatism [6, 22, 25], and particularly by Lewis’s precept that the conceptual frames used to grasp and organize particular experiences are hypothetical in nature, and constantly subject to revision according to pragmatic needs [25].^9^ A change of conceptual framework will elicit a new configuration of appearances, which can in turn become new resources for and objects of intervention. Put differently, if our models of the world are never completely isomorphic with their objects, it follows that the world re-described can be the world known differently. Indeed, it is this insight that underlies a signature component of improvement methodologies, the PDSA (Plan-Do-Study-Act) cycles whereby component processes of a system are iteratively intervened upon for purposes of continuous improvement [25].

## In Conclusion: Improvement Science as a Para-Paradigm?

What does this brief and partial survey of currents informing the development of improvement science tell us about its ecumenicalism as a field? Here I have drawn upon a range of disciplinary perspectives to suggest that improvement science is informed by two tendencies that are in strong tension with one another. One tendency emphasizes the advantages and even necessity of what I’ve glossed here as gap closure: the drive to close spaces of misalignment (for example, between word and world), and to enforce clear and unambiguous boundaries (of language, of disciplines, and possibly of improvement science as a paradigm). This perspective gained coherence and force in tandem with the modern development of the natural sciences, and is today largely congruent with common sense within both clinical science and the natural sciences. Another perspective recognizes the benefits that flow from a less rigid insistence on alignment and gap closure, and counsels attentiveness to the partial and provisional character of our descriptions, categories, and models. This view is consonant with some of the philosophical precepts that inform improvement science, and has gained further elaboration in recent years in conjunction with what’s been referred to as the “complexity turn” [31].

Can two such starkly contrasting positions *both* be held within improvement science? Can *this* gap be bridged, or is such an exercise of alignment even necessary? I believe that the two positions can both be held, and for reasons that might also provide grounds for considering improvement science itself to be an ecumenical enterprise, confidently receptive to a host of disciplinary approaches. To indicate why I believe this to be the case, consider one possible argument to the contrary. It might be argued that the distance between these two perspectives is so great, their differences so fundamental, that they cannot both be accommodated within the same paradigm. In this case we might say that the “gap” between them is an unbridgeable chasm; that they are incommensurable. Indeed, it might be claimed that to assert otherwise would be to disregard the kind of internal consistency and coherence we tend to associate with a dominant paradigm and the conduct of normal science. Such a concern seems to inform efforts to cast improvement science as a paradigm *in statu nascendi*: as on its way to paradigmatic consolidation and normalization, but still falling short of consensus on its exact definition [21]. Such a view might urge that improvement science should be vigilantly defended against incursions by anything deemed to be insufficiently scientific.

Yet invocation of the language of paradigms also suggests another possibility. On one hand the tension I have described within improvement science might be seen as a classical instance of paradigmatic incommensurability. But in the case of improvement science the question of dialogue between supposedly incommensurable positions has an added twist. This is because the second position I have sketched (the pragmatic, complexity inflected view) itself contains something of an account of incommensurability: in acknowledging that different perceptual frames will capture different aspects of an endlessly complex world, and in evincing comfort with the fact that such different renderings need not necessarily (as opposed to strategically) be reconciled, the second tendency seems somehow prophylactically protected against implication in the snares of incommensurability. In much the same way that a spider can nimbly glide along the filaments of its own web without getting caught, so too might we think of the second tendency I have described as being able to weave deftly among different positions, without becoming ensnared in a viciously unending circle of incommensurability. Absent the imperative to mind the gap, the vicious circle becomes one of virtue: an indication of plenitude rather than of epistemological infirmity.

As a final genealogical observation, it is worth noting that Kuhn’s own conception of paradigms was based on convictions that are strikingly congruent with those I have described in association with the pragmatism of C.I. Lewis, and with certain precepts associated with complexity theory.^10^ For Kuhn the explanatory power of a paradigm was never absolute, but always relative to the perceptual and linguistic frameworks that necessarily delimited and simplified a universe that was otherwise “overwhelmingly dense, complex, and filled with epistemic possibilities” [1]. According to Kuhn this boundless complexity had to be “cut” to be made manageable:

…natural language provides a finite means of mediating an infinitely complex universe…. Put more accurately—we in fact live in a world much more complex than our language admits. If we are to act in it, we must simplify it, and our choice of a particular manner of simplification (a cut) is pragmatically determined and is embodied in our language [28].

Bearing in mind this reflexive dimension of improvement science—the fact that it seems well-equipped to provide an account of its own internal inconsistencies—perhaps it is better considered as something other than a paradigm as conventionally understood in the natural sciences. Such a view would in no way detract from the power of improvement science; rather it would comprise a clear-eyed acknowledgement of the field’s *defacto* richness and heterogeneity, both in terms of its theoretical foundations and interdisciplinary influences and methods, and in relation to its historical development as described here. Keeping in mind the field’s heterogeneity, perhaps improvement science is better considered as a para-paradigm: as a vital adjunct to clinical science, but one which resists modelling on the template of the natural sciences. If the immense power of clinical science derives from scrupulous obedience to the imperative to “mind the gap”, improvement science as a supportive adjunct could be said to draw a good deal of *its* strength from a principled and reflexive ability to selectively *not* mind the gap.

If such a conclusion would seem to tilt the scale in a manner which runs against the grain of perfectly balanced ecumenicalism between the two tendencies described here, it nonetheless seems to be justified. Sociologist Andrew Abbott [1] has suggested that because paradigms in the social sciences and humanities are not guided by norms of “progress” in the same way that paradigms in the natural sciences are, they require other normative criteria for judgment. Abbott makes a good case for “plenitude” and “plurality” as normative criteria for knowledge in the social sciences and humanities. Given that improvement science bears the genealogical imprint of both the natural and the social sciences, plentitude and plurality would seem to be good normative criteria for it, too. Such an ecumenical embrace of plenitude and plurality accommodates the dual spirits of scientism and pragmatism that animate improvement science, and can provide a sound and stable foundation for the field’s continued growth and future flourishing. In practice this would entail acceptance of the field’s genealogical hybridity, and recognition that each of the impulses described here—that of gap-closure and gap-tolerance, has its place and uses. Such ecumenicalism can in turn both support and reciprocally derive strength from the practice of reflexivity on the part of practitioners of every stripe: reflexivity concerning the rich and multi-faceted philosophical strands that inform improvement science, and reflexive professional practice that combines the rigor of scientism with the flexibility of pragmatism.

## Footnotes

1 Three of the founding figures of improvement science—Walter Shewhart (1891–1962), W. Edwards Deming (1900–1993), and Joseph Juran (1904–2008)—were American statisticians and engineers, variously active in industrial manufacturing, military production during the Second World War, and the post-War reconstruction of Japanese industry.

2 The global and recursive character of quality improvement was formalized with the adoption in 1987 of ISO 9000, an international series of standards for the certification of both quality and the means by which it will be assured.

3 These include Lean, Six Sigma, Total Quality Management, and the Toyota Production System.

4 The Institute of Medicine’s two landmark reports, *To Err is Human* and *Crossing the Quality Chasm* were published in 1999 and 2001 respectively, and according to Martin Marshall the term “improvement science” itself had been in use in medicine for at least a decade prior to that [20].

5 Complexity theory is not easily defined, although a serviceable definition is as follows: A set of concepts that attempts to explain complex phenomenon not explainable by traditional (mechanistic) theories. It integrates ideas derived from chaos theory, cognitive psychology, computer science, evolutionary biology, general systems theory, fuzzy logic, information theory, and other related fields to deal with the natural and artificial systems as they are, and not by simplifying them (breaking them down into their constituent parts). It recognizes that complex behavior emerges from a few simple rules, and that all complex systems are networks of many interdependent parts which interact according to those rules. (http://www.businessdictionary.com/definition/complexity-theory.html accessed August 7, 2016).

6 Other examples of complex adaptive systems include the human body’s immune system; a colony of social insects such as termites or ants; and the stock market.

7 Thrift has trenchantly described how the enlistment of “complexity theory as metaphor”, along with the explicit promotion of a non-referential theory of language, were part of a new style of management associated with neoliberal restructuring of public services that was underway in the 1980s and 1990s. [29] The role of “complexity as metaphor” in cultivating proactive, responsibility-bearing subjects of the sort normatively valorized by neoliberalism is an undoubtedly important and widely ramifying phenomenon, but well beyond the scope of the present piece and this special issue’s concern with improvement science.

8 Although Peirce left Harvard in 1879 for employment at Johns Hopkins University, he remained an active and influential figure in the Harvard-based circle of pragmatist philosophers sometimes referred to as the “Metaphysical Club” [24].

9 Recalling Nelson Goodman’s aforementioned philosophical concern with the misleading potential of ascriptions of similarity on account of their inevitably partial basis, it is worth noting that he, too, worked in the shadow of the original pragmatists, completing his PhD at Harvard in 1941 under the supervision of C.I. Lewis. [17].

10 During his intellectually formative years at Harvard (1940–1956) a young Thomas Kuhn may have encountered both C.I. Lewis, who taught there from 1920 to 1953, and Nelson Goodman, who completed his Ph.D. at Harvard in 1941. With his election to Harvard’s Society of Fellows, Kuhn would have almost certainly encountered one of Lewis’s most renowned students, the philosopher W.V. Quine. (http://plato.stanford.edu/entries/thomas-kuhn/).
